# Optical Surface Transformation: Changing the optical surface by homogeneous optic-null medium at will

**DOI:** 10.1038/srep16032

**Published:** 2015-10-30

**Authors:** Fei Sun, Sailing He

**Affiliations:** 1 State Key Laboratory of Modern Optical Instrumentations, Centre for Optical and Electromagnetic Research, JORCEP, East Building #5,Zijingang Campus, Zhejiang University, Hangzhou 310058, China; 2 Department of Electromagnetic Engineering, School of Electrical Engineering, Royal Institute of Technology (KTH), S-100 44 Stockholm, Sweden

## Abstract

A new theory on designing electromagnetic/optical devices is proposed, namely, an optical surface transformation (OST). One arbitrary surface can establish the corresponding relationship with another surface entirely optically with an optic-null medium (ONM), (i.e. the electromagnetic wave propagates from one surface to its equivalent surface without any phase delay). Many novel optical devices can be designed by an OST with the help of an ONM. Compared with traditional devices designed by Transformation Optics, our optical surface-reshaping devices have two main advantages. Firstly, the design process is very simple (i.e. we do not need to consider any mathematics on how to make a coordinate transformation, and what we need to do is simply to design the shapes of the input and the output surfaces of the devices). Secondly, we only need one homogeneous anisotropic medium to realize all devices designed by this method. Our method will explore a new way to design novel optical devices without considering any coordinate transformations.

Transformation optics (TO) is a powerful theoretical tool for designing novel electromagnetic/optical devices by a coordinate transformation method^1^,^2^,^3^,^4^. With the help of coordinate transformations, we can establish a corresponding relation between two spaces: the one is a virtual space referred as the reference space, and the other is the real space. Based on the form-invariance of Maxwell’s equations under coordinate transformations, a corresponding relationship related with the specific coordinate transformation can also be established between the electromagnetic fields and media in two spaces. Many novel devices have been designed by TO, including invisibility cloaks^1^,^5^,^6^,^7^,^8^, PEC reshapers^9^,^10^, beam splitters^11^,^12^, beam compressors^13^,^14^, wave front convertors^15^,^16^, carpet cloaks^17^,^18^,^19^, novel lenses^20^,^21^, optical illusion devices^22^,^23^ etc. The idea of controlling fields by the coordinate transformation method has also been extended to other physical fields, e.g. DC magnetic field^24^,^25^, thermal field^26^,^27^, and acoustic field^28^,^29^. In recent years, there are still many studies on the theory of TO, e.g., the field transformation method^30^,^31^,^32^, the triple space-time transformation^33^, the conformal/quasi-conformal transformation^34^,^35^,^36^, the complex transformation^37^,^38^, etc. In all these methods, people always need to make some mathematical calculations during the designing process, which makes it difficult to be applied directly in engineering. Furthermore, the media designed by TO and other methods are often very complicated, and have to be simplified before being made into metamaterials (i.e. some artificial media whose electromagnetic properties are mainly determined by the artificial unit structure instead of the chemical component^39^). Although conformal/quasi-conformal mappings can help eliminate the anisotropy, the devices designed by conformal/quasi-conformal mappings are still inhomogeneous, making them difficult to realize.

In this work, we propose a novel method (called Optical Surface Transformation (OST)) to design optical/electromagnetic devices without making any mathematical calculations. All we need to do is simply to design the shapes of the input and the output surface of the devices. The optical designing process becomes much simpler. Another important feature of the devices designed by our method OST is that we only need one homogeneous medium to realize them. After some theoretical calculations, we find that this medium is an optic-null medium (ONM)^40^ (also called an optic void^41^ or a null-space medium^21^,^42^ in other studies).

We should note that previous studies on the ONM are conducted in a Cartesian coordinate or a cylindrical coordinate system^40^,^41^,^42^,^43^,^44^. A slab region in a Cartesian coordinate system or a cylindrical ring in a cylindrical coordinate system in the real space is completely compressed into a plane or a cylindrical surface in the reference space. The corresponding medium in the real space is an optic-null medium (a highly anisotropic homogenous medium), which has been utilized to realize hyper-lenses for super-resolution imaging^43^. For a DC magnetic field, we can also design such a medium for a magnetic hose^44^ or DC magnetic concentrator^45^. In this work, we explore some other applications of the ONM that can help us to achieve an OST and design many other novel devices that need some irregularly shaped ONM.

## Results.

### Surface Projecting by ONM

**Figure 1:**
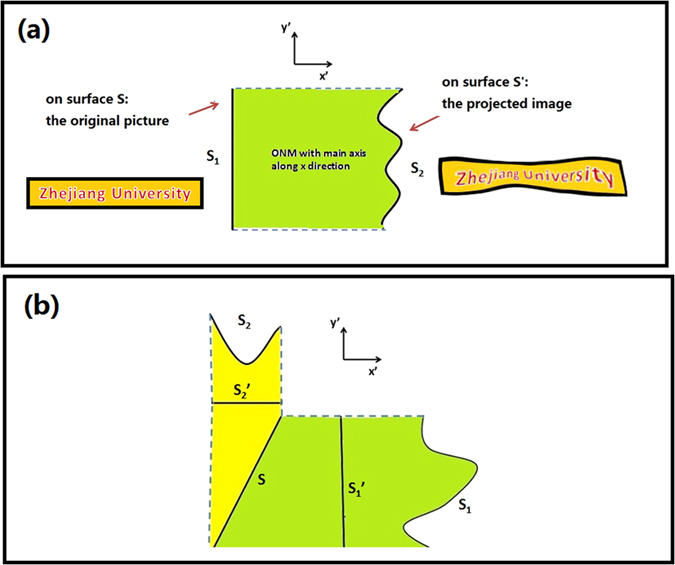
(**a**) The basic idea of the OST: Surface *S*_1_ (a plane) and *S*_2_ (a curved surface) are connected by the optic-null medium (ONM). Thus, the information (e.g. a picture) on surface *S*_1_ can be projected to surface *S*_2_ optically (a distorted picture). *S*_1_ and *S*_2_ can be treated as input and output surfaces of the device, respectively. Note that *S*_1_ and *S*_2_ can be arbitrarily shaped surfaces (*S*_1_ is not necessarily a plane). (**b**) A general outline for making two arbitrarily shaped surfaces *S*_1_ and *S*_2_ optically equivalent. We first project the curved surface *S*_1_ to a plane *S*_1_’ along the *x’* direction, and project the curved surface *S*_2_ to another plane *S*_2_’ along the *y’* direction. Next we keep on projecting *S*_1_’ along the *x’* direction and *S*_2_’ along the *y’* direction, and obtain a common plane *S* that is the diagonal of the rectangle outlined by the shifted *S*_1_’ and *S*_2_’. This figure is drawn by one author (Fei Sun).

The basic idea of our method is shown in Fig. 1(a): Two arbitrary shaped surfaces can be linked by a single homogenous anisotropic medium (the ONM). In this case, each point on surface *S*_1_ gets a corresponding relationship with another point on surface *S*_2_. If we set a picture on surface *S*_1_, we will get its imaging on surface *S*_2_. The ONM performs like a projecting transformation (e.g. projecting along the *x’* direction in Fig. 1) from *S*_1_ to *S*_2_. The material parameters of an ONM can be given by:


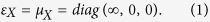



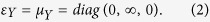


In practice, we just need to make the homogenous medium highly anisotropic with the permittivity and permeability extremely large along one direction and nearly zero along other orthogonal directions. There have been many studies on how to realize such an ONM (e.g. a holed metallic plate with fractal-like apertures^40^ or a metallic slit array satisfying the Fabry-Pérot resonances condition^41^). Note that the label ‘*X*’ or ‘*Y*’ means the main axis of this ONM is along *x’* or *y’* direction, i.e. the projecting transformation is along the *x’* or *y’* direction, respectively. Similarly the main axis of the ONM can be in any other directions, e.g. in the radial direction (in this case the permittivity and permeability of the ONM is extremely large along the radial direction and nearly zero along other orthogonal directions, and its function is to project surfaces along the radial direction).

**Figure 2:**
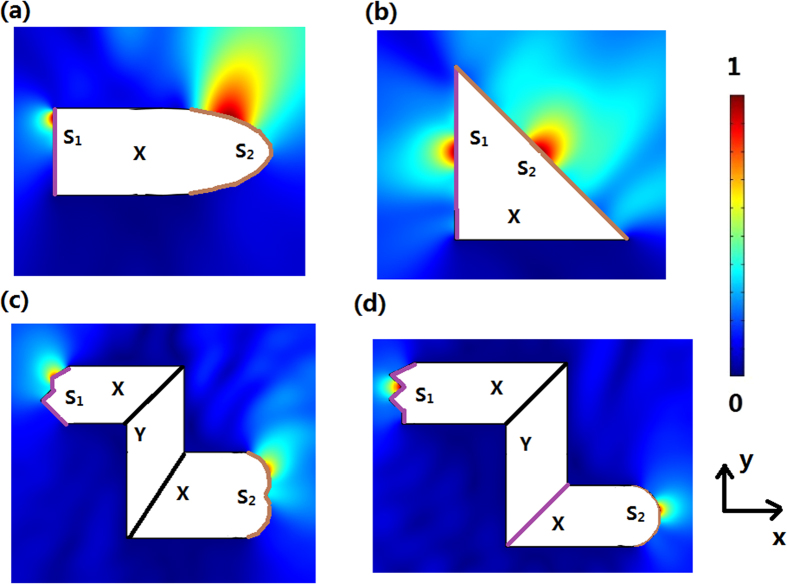
The 2D finite element method (FEM) simulation results: the absolute value of the normalized electric field’s *z* component for the TE wave case. We set a line current source at the input surface *S*_1_ (purple line). The white region is filled with the ONM. We get an image of the line current at the output surface *S*_2_ (brown line). For (**a**–**d**), we choose different shapes for the input and output surfaces. The white regions labeled ‘*X*’ or ‘*Y*’ is the ONM described by Eq. (1) or (2), respectively, throughout the whole paper.

Two arbitrarily shaped surfaces in a 2D space can always have a common projecting plane by projecting along *x’* and *y’* direction. As shown in Fig. 1(b), we can first project the arbitrarily shaped surfaces *S*_1_ and *S*_2_ along *x’* and *y’* directions to plane *S*_1_’ and *S*_2_’, respectively. Then we can further project *S*_1_’ and *S*_2_’ along *x’* and *y’* directions, respectively, to a common surface *S,* which is the diagonal surface of *S*_1_’ and *S*_2_’. Now we have made two arbitrarily shaped surfaces *S*_1_ and *S*_2_ linked by projecting along *x’* and *y’* directions, respectively. In the method section, we will show that the ONM with its main axis in *x’* and *y’* directions can perform like such a projecting transformation along *x’* and *y’* directions, respectively, by TO. From the perspective of TO, two arbitrary surfaces *S*_1_ and *S*_2_ connected by the ONM in Fig. 1(b) correspond to the same surface in the reference space. This means that such two surfaces will perform equivalently to the optical wave (i.e. there will be no phase delay when the wave propagates from *S*_1_ to *S*_2_). This also means that any optical information on surface *S*_1_ can be exactly transformed to surface *S*_2_. For example, if we put a point source on surface *S*_1_, we will get its image at the corresponding point on surface *S*_2_ (see Fig. 2).

Note that all media appearing later in this paper are ONM described by Eq. (1) or (2), which are labeled, respectively, by ‘*X*’ or ‘*Y*’ to indicate that the main axis is along the *x’* or *y’* direction.

### Transform the focusing plane of the traditional lens at will

Optical conformal mapping (CM) or quasi-conformal mapping (QCM) can be utilized to transform the circular focusing plane of the Luneburg lens or Maxwell’s fish eye lens to a plane^46^,^47^,^48^. However, we have to numerically generate the quasi-conformal spatial transformation first, and then design an inhomogeneous medium to realize it. If we use the idea of the OST, we can simply transform the circular focusing plane of a traditional Luneburg lens or Maxwell’s fish eye lens to a plane by setting a homogeneous ONM aside the lens. The designing process is very simple: the input surface *S*_1_ of the optical surface-reshaping device is the original focusing plane of the traditional lens; the output surface *S*_2_ of the optical surface-reshaping device is the new focusing plane with the desired shape. The medium connecting the two surfaces *S*_1_ and *S*_2_ is the optic-null medium described by Eq. (1) or (2).

**Figure 3:**
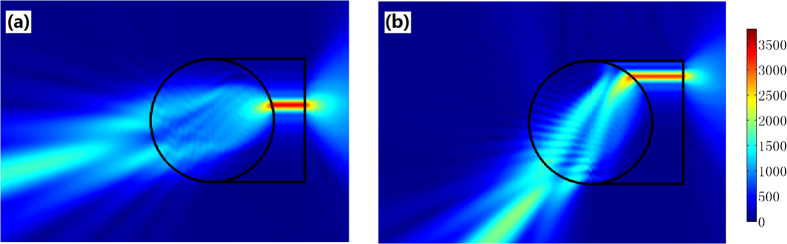
The 2D FEM simulation results: the absolute value of the electric field’s z component (the TE wave case). We set an optical surface-reshaping device (the region around the circle) around a traditional Luneburg lens (the circle), so that the focusing plane of the whole system is converted into a plane. From (**a**,**b**), we shift the position of a line current with unit amplitude 1A at the focusing plane from the center to the edge. The direction of the output beam changes accordingly.

**Figure 4:**
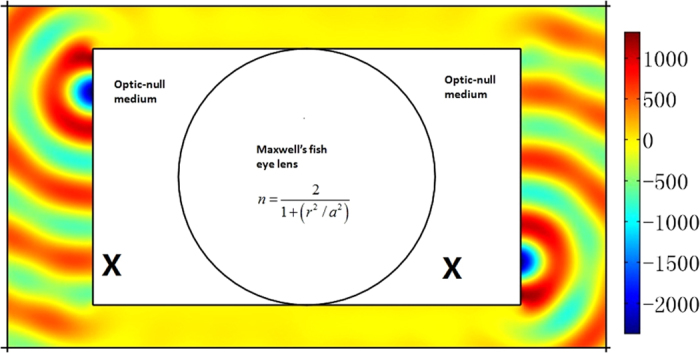
The 2D FEM simulation results: we plot the *z* component of the electric field for TE wave case. We set a line current with unit amplitude 1A at the left side of the whole system. An image is obtained at the right side of the whole system. The whole imaging system (the white region) is composed by setting two ONM aside a traditional Maxwell’s fish eye lens.

As shown in Fig. 3, we put an optical surface-reshaping device (*S*_1_ is a semi-circle and *S*_2_ is a plane) aside a traditional Luneburg lens (the circular shaped region). The optical surface-reshaping device is filled with the optic-null medium whose main axis is along the *x’* direction. In this case, the focusing surface of the Luneburg lens is transformed from a half-circle to a plane. Similarly we can set two optic-null media aside the Maxwell’s fish eye lens to transform two circular focusing planes into two planes (see Fig. 4).

### The wave front convertor

**Figure 5:**
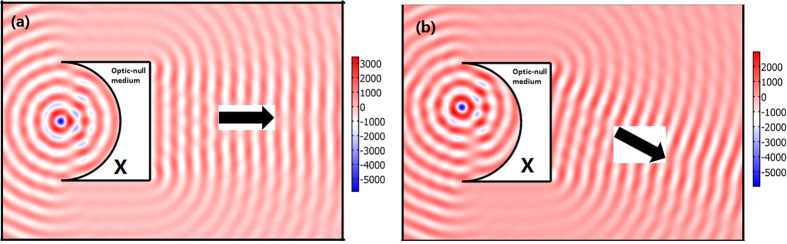
The 2D FEM simulation results: we plot the electric field’s *z* component for the TE wave case. A line current with unit amplitude 1A is set on the center or off the center of the input circle of our device (the white region). The input surface and output surface of our device are the circle and plane, respectively. The wave front of the output beam beyond our device is a plane. If we change the position of the line current, the direction of the output beam (labeled by the black arrow) changes accordingly.

**Figure 6:**
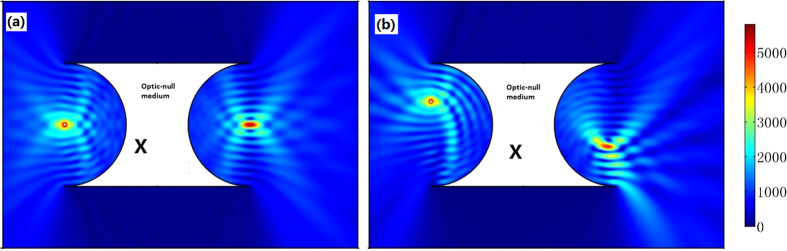
The 2D FEM simulation results: we plot the absolute value of the electric field’s *z* component for the TE wave case. We set a line current with unit amplitude 1A producing a diverging cylindrical wave at the left side of our optical surface-reshaping device (the white region) whose input and output surfaces are both circles. The line current is on the center or off the center of the input circle of our device for (**a**,**b**), respectively. A converging cylindrical wave is obtained beyond the output surface of our device.

As the input surface and output surface of our optical surface-reshaping device correspond to the same surface in the reference space, an incident wave whose wave front has the same shape as the input surface of the device will be transformed to an output wave whose wave front has the same shape as the output surface of the device. The designing process of the wave front convertor is simply to design the shape of the input and output surfaces of the device. In addition, these devices can be simply realized by a single homogenous medium (the ONM described by Eqs. (1) or (2)). For examples, we can easily choose the input surface as a cylindrical surface and the output surface as a plane to perform like a cylindrical-plane wave convertor (e.g. transforming a cylindrical wave produced by a line current to a plane wave, see Fig. 5). If we want to transform a diverging cylindrical wave produced by a line current to a converging cylindrical wave, we can also design an optical surface-reshaping device whose input and output surfaces are both cylinders (see Fig. 6).

### Other applications

**Figure 7:**
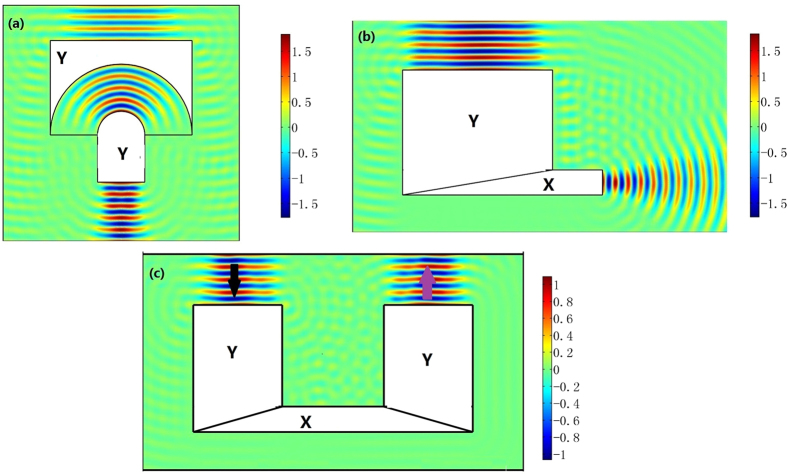
The 2D FEM simulation results: we plot the electric field’s *z* component for the TE wave case. (**a**) A beam compressor: a Gaussian beam with half waist width 5.5λ_0_ is imposed onto two optical surface-reshaping devices from top to bottom. At the output surface of the second device, the half width of the output beam reduces to 2λ_0_. (**b**) Another beam compressor: the half waist width of the incident Gaussian beam is 5.5λ_0_. The output beam is compressed to a beam with half width λ_0_ at the output surface. As the size of the output beam approaches the size of the wavelength, it quickly diverges beyond the output surface of the device. (**c**) A beam redirector: a Gaussian beam is incident from the top (the black arrow) onto the device and is redirected back (the purple arrow).

Our optical surface-reshaping device can also be utilized as a beam controller (e.g. a beam compressor, expander, splitter, etc.). For example, we can first use one surface-reshaper to transform a Gaussian beam with a wide width into a converging cylindrical wave, and then use another surface-reshaper to transform this converging wave to a Gaussian beam with a narrow width (see Fig. 7(a)). We can also simply choose the input surface and output surface of our optical surface-reshaping device as two planes with different areas to make a beam compressor/expander (see Fig. 7(b)). A beam redirection device can also be designed by our optical surface-reshaping device (see Fig. 7(c)). Although these devices can also be designed by TO (e.g. the finite embedded transformation^11^,^12^,^13^,^14^), the required materials are very complex (e.g. inhomogeneous anisotropic materials). The devices based on OST here can eliminate the inhomogeneity requirement.

**Figure 8:**
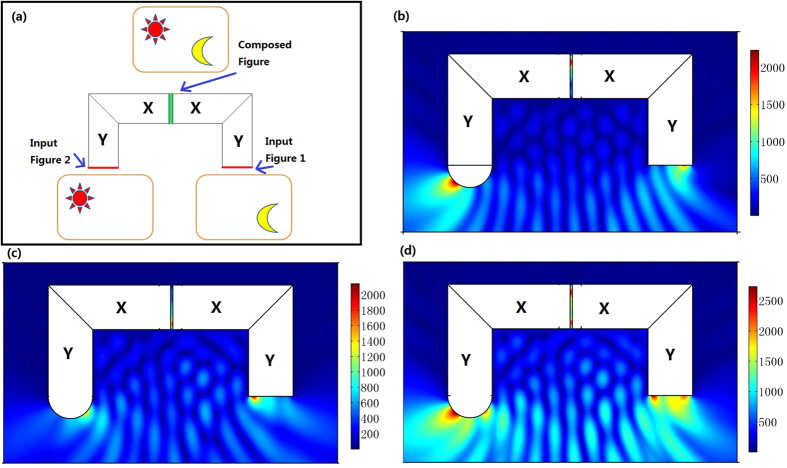
(**a**) The basic idea of the optical figure combination: we place two figures at two input surfaces of our surface-reshaping devices, and put the two output surfaces very closely. In this case, we can obtain a composed figure entirely optically in the air gap region between the two output surfaces. (**b**–**d**) The 2D FEM simulation results: we plot the absolute value of the electric field’s *z* component. (**b**) We only set a point source at the left input surface (note the shape of the input surface here is a circle). (**c**) We only set a point source at the right input surface. (**d**) We set simultaneously one point source at the left input surface and another point source at the right input surface. In this case, we obtain two point images in the small center air gap region between the two output surfaces. Figure 8(a) is drawn by one author (Fei Sun).

As the input and output surfaces of our optical surface-reshaping device correspond to the same surface in the reference space, we can achieve an all-optical pattern combination by the cascade connection of our devices. Figure 8(a) shows the basic idea of this application. In this way, we can directly combine two patterns optically (traditionally we need to use some digital pattern processing technique). Figure 8(b–d) give examples in which we combine the images of two point sources in two patterns into one single surface. With the help of our optical surface-reshaping device, we can simple carry out the optical pattern process entirely optically, which includes combination, scaling, splitting, and projecting. This method may pioneer a new way to all-optical calculation, optical message processing, and optical computing systems.

We can also design some devices that can control the polarization of the electromagnetic waves, e.g. polarization splitters. Our method can be extended to a 3D space, though it may be a bit complicated. In the 3D case, more devices can be designed to e.g. control, transform or convert the polarization and these can be for future work.

**Figure 9:**
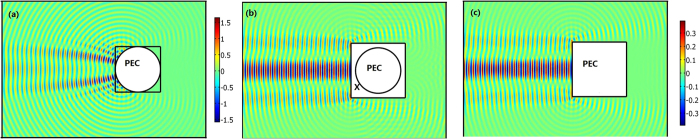
The 2D FEM simulation results: we plot the electric field’s *z* component for the TE wave case. A Gaussian beam with half-waist width 4λ_0_ is incident from the −*x’* direction onto a PEC: (**a**) a cylinder with radius 5λ_0_, (**b**) the cylinder composed by the optic-null medium with its main axis in the *x’* direction, and (**c**) a rectangle with the same size as the composed object in (**b**). The scattering features of (**b**,**c**) are exactly the same.

Our surface-reshaping device can also change the geometry of a PEC to an arbitrary shape, e.g. the scattering feature of a cylinder PEC is transformed to a rectangular PEC (see Fig. 9).

## Discussions

The designing process by OST can be summarized as: first we need to determine the shape of the input surface and the output surface according to a specific application. For example if we want to transform a cylindrical wave to a spherical wave, the shape of the input and output surfaces of the device should be cylindrical and spherical, respectively. If we want to project a picture on a plane to a curved surface, the input and output surfaces of our device should be chosen as the plane and curved surfaces, respectively. Secondly, we need to find a direction that can project the input surface to the output surface. That direction is also the main axis of the ONM filled inside the device. Note that the projecting may be achieved by many times in many different directions. In this case, the device is composed by the combination of many ONMs with different main axes.

There are many papers on studying optical void medium^40^,^41^,^42^,^43^,^44^,^45^. All previous papers are focused on how to derive the optical void by transformation optics for different applications (e.g. perfect lenses^40^,^43^, concentrators^41^,^45^). All previous studies still need a mathematical calculation by the standard formula of transformation optics. In our manuscript, we first proposed the idea to design novel electromagnetic/optical devices by using the equivalent surfaces without any mathematical calculation, which is different from the previous studies on the optical void.

There are already many studies on how to realize such a highly anisotropic material (the optic-null medium^40^,^41^). A metallic slit array that satisfies the Fabry-Pérot (FP) resonances has been utilized to realize the ONM, which works in a series of frequency bands around the FP resonances^41^. The loss of the metal doesn’t influence the performance of devices composed by the ONM, which can also be an effective way to realize experimentally the novel devices designed by our proposed OST.

Although the beam redirector in Fig. 7 can also be realized by near-zero-epsilon materials^49^, many other devices designed by OST and realized by ONM simply cannot be realized by near-zero-epsilon materials, e.g., if we replace the ONM in Fig. 2 with a near-zero-epsilon material, no such point-to-point imaging function can be achieved. There is a main axis in an ONM that can be treated as a tunnel/channel linking two equivalent surfaces. Two surfaces of arbitrary shapes linked by zero-epsilon materials cannot perform equivalently, which is essentially different from the ONM. Our method can be extended to a 3D space, though it may be a bit complicated. In the 3D case, more devices can be designed to e.g. control, transform or convert the polarization and these can be for future work.

Traditional design of TO first requires a proper coordinate transformation either analytically or numerically, and the transformation medium is often inhomogeneous (i.e. gradient control is required). In this paper, we propose the idea of OST that can help design novel optical devices easier. Most devices designed by the coordinate transformation method (e.g. wave front convertors, PEC reshapers, beam splitters, beam compressors, etc.) can be designed by the OST. The main advantage of OST can be summarized in two points. Firstly, the design process is very simple, only requiring the design of the input and output surfaces of our devices. There is no need to consider what kind of coordinate transformation can be used. Secondly, we only need one homogeneous anisotropic material (i.e. ONM) to realize our optical surface-reshaping device. Our all-optical surface-reshaping device can have many potential applications in all-optical calculation, all-optical image processes, optical image projecting, beam controlling, wave front reshaping, novel lenses, etc.

## Method

We will show that the ONM with its main axis along *x’* and *y’* directions can make a projection transformation along *x’* and *y’*, respectively by TO^1^,^2^,^3^,^4^. We will also explain how to make two arbitrary surfaces in the real space corresponded to the same surface in the reference space by TO. We assume that the quantities with and without primes indicate the quantities in the real and reference space, respectively. For simplicity we only consider the 2D case (i.e., it is infinitely long in the *z’* direction) in the paper, but the idea can be directly extended to the 3D case. In order to make two arbitrarily shaped surfaces (e.g. *S*_1_ and *S*_2_) in the real space correspond to the same surface in the reference space, we first establish the corresponding relationship between each arbitrary surface (*S*_i_, *i* = 1, 2) and its plane (*S*_i_’, *i* = 1, 2), respectively (see Fig. 1(b)). The next step is to link two planes by another common plane *S*.

**Figure 10:**
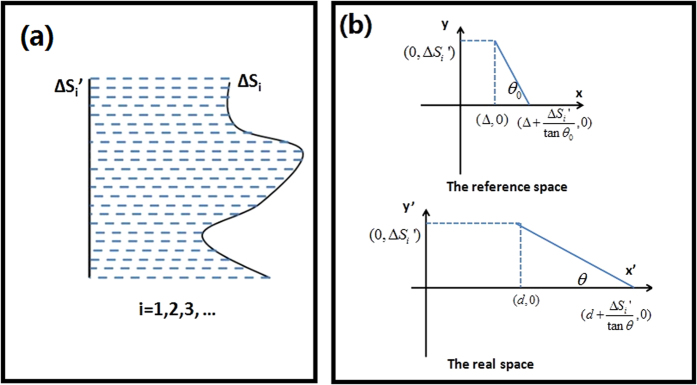
(**a**) An arbitrarily shaped surface is divided into many small plane elements Δ*S*_*i*_. Each element is projected along the horizontal direction and is linked with a small element of a plane Δ*S*_*i*_’. A corresponding relation between the whole arbitrarily shaped surface and a plane is established in this way. (**b**) The two small plane elements Δ*S*_i_ and Δ*S*_i_’ in the real space correspond to one common small plane element Δ*S*_i_’ in the reference space when *θ*_0_ → 90^o^ and ∆ → 0.

We first show how to make the arbitrarily shaped surface *S*_1_ and the plane *S*_1_’ (linked by the green medium in Fig. 1(b)) corresponded to the same single plane in the reference space. When the arbitrarily shaped surface *S*_1_ is given, we can project it along the *x’* direction to the plane *S*_1_’, and then we can divide *S*_1_ and *S*_1_’ into many small plane elements (see Fig. 10(a)). Next we want to establish the corresponding relationship between each divided small plane with different slopes Δ*S*_i_ and its projected small plane Δ*S*_i_’ (i = 1, 2, 3…). In other words, we need to find a coordinate transformation to make two small planes Δ*S*_i_ and Δ*S*_i_’ in the real space correspond to the same small plane in the reference space. The coordinate transformation is given by (see the relation in Fig. 10(b)):


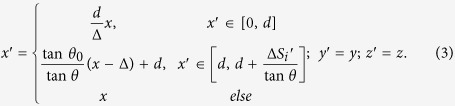


The relative permittivity and permeability in each region can be calculated with the help of TO^1^,^2^,^3^,^4^:


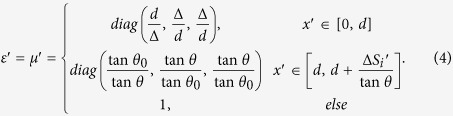


Note that *θ* and *d* can have arbitrary values. When *θ*_0_ → 90^o^ and ∆ → 0, the trapezoid region in the reference space will be reduced to a single surface element. In this case the medium in the trapezoid region of the real space can be obtained from Eq. (4) by taking *θ*_0_ → 90^o^ and ∆ → 0:


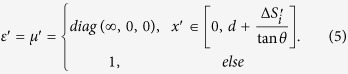


As we can see from Eq. (5), the medium in the trapezoid region of the real space is the ONM with the main axis along the *x’* direction. The green region between *S*_1_ and *S*_1_’ in Fig. 1(b) can be divided into many small trapezoid regions (see Fig. 10(a)) along the *x’* direction. If we fill the ONM whose relative permittivity and permeability are given in Eq. (5) in each small trapezoid region, each small trapezoid region corresponds to a single surface in the reference space. Hence surfaces *S*_1_ and *S*_1_’ linked by the green ONM correspond to a common single surface in the reference space. Note that the medium that links *S*_1_’ and *S* is exactly the same as the medium that links *S*_1_ and *S*_1_’ as they are both obtained by projecting along the *x*’ direction. Surface *S* can be treated as the special case that surface *S*_1_ is chosen as a plane.

Now we show that *S*_1_ and *S*, linked by the green ONM in Fig. 1(b) are equivalent surfaces in the real space. The green ONM which has infinitely large permittivity and permeability along the *x’* direction and nearly zero permittivity and permeability along other orthogonal directions, is defined as the ONM of the main axis along the *x’* direction in Eq. (1). Similarly the yellow optic-null medium in Fig. 1(b) that links surfaces *S*_2_ and *S* can also be calculated by projecting *S*_2_ to *S*_2_’ and *S* to *S*_2_’ along the *y’* direction (no essential difference in mathematics). The yellow ONM whose main axis is along the *y’* direction is described in Eq. (2). The surfaces *S*_2_ and *S* linked by the yellow ONM whose main axis is along the *y’* direction corresponds to the same single surface in the reference medium, which means that *S*_2_ and *S* are optically equivalent surfaces.

Now we can summarize the above result: surfaces *S*_1_ and *S* linked by an ONM with the main axis along the *x’* direction corresponds to the same single surface in the reference space; surfaces *S* and *S*_2_ linked by an ONM with the main axis along the *y’* direction also corresponds to the same surface in the reference space. Now we make two arbitrary surfaces *S*_1_ and *S*_2_ corresponding to the same single surface in the reference space by the ONM. This means that surfaces *S*_1_ and *S*_2_ are equivalent surfaces (e.g. any optical information on surface *S*_1_ can be exactly transformed to surface *S*_2_), which has been verified by many examples in the main text.

## Additional Information

**How to cite this article**: Sun, F. and He, S. Optical Surface Transformation: Changing the optical surface by homogeneous optic-null medium at will. *Sci. Rep.* **5**, 16032; doi: 10.1038/srep16032 (2015).
